# Efficient Virus-Induced Gene Silencing in *Solanum rostratum*

**DOI:** 10.1371/journal.pone.0156228

**Published:** 2016-06-03

**Authors:** Lan-Huan Meng, Rui-Heng Wang, Ben-Zhong Zhu, Hong-Liang Zhu, Yun-Bo Luo, Da-Qi Fu

**Affiliations:** Laboratory of Food Biotechnology, College of Food Science and Nutritional Engineering, China Agricultural University, No. 17 Qinghua Donglu Road, Haidian District, Beijing, 100083, China; Mediterranean Agronomic Institute at Chania, GREECE

## Abstract

*Solanum rostratum* is a “super weed” that grows fast, is widespread, and produces the toxin solanine, which is harmful to both humans and other animals. To our knowledge, no study has focused on its molecular biology owing to the lack of available transgenic methods and sequence information for *S*. *rostratum*. Virus-induced gene silencing (VIGS) is a powerful tool for the study of gene function in plants; therefore, in the present study, we aimed to establish tobacco rattle virus (TRV)-derived VIGS in *S*. *rostratum*. The genes for phytoene desaturase (*PDS*) and Chlorophyll H subunit (*ChlH*) of magnesium protoporphyrin chelatase were cloned from *S*. *rostratum* and used as reporters of gene silencing. It was shown that high-efficiency VIGS can be achieved in the leaves, flowers, and fruit of *S*. *rostratum*. Moreover, based on our comparison of three different types of infection methods, true leaf infection was found to be more efficient than cotyledon and sprout infiltration in long-term VIGS in multiple plant organs. In conclusion, the VIGS technology and tomato genomic sequences can be used in the future to study gene function in *S*. *rostratum*.

## Introduction

*Solanum rostratum *is a native North American plant species whose range extends from central Mexico northward across the Great Plains of the United States [1]. Its seeds can grow in extreme drought conditions, rapidly spread in a variety of environments, and produce the toxic glycoalkaloid compound solanine that is harmful to humans and other animals [2]. Many countries have invested in multiple efforts to control the hazards posed by this plant species. To date, research at the molecular level is limited in *S*. *rostratum* because of the lack of transgenic techniques and the availability of related genome sequences [3]. Improving the genetic information and molecular tools in *S*. *rostratum* would drive research in controlling/cultivating this species forward. In addition, confirming the molecular and physiological information that is shared among members of the *Solanaceae* family will provide new directions for the research of *S*. *rostratum*.

*S*. *rostratum* belongs to the *Solanaceae* family, among which tobacco and tomato serve as model organisms. In these model systems, virus-induced gene silencing (VIGS) has been successfully applied to tomato [4], potato [5], and *N*. *benthamiana* [6], among others. If the gene sequences and VIGS technology of tomato can be applied to *S*. *rostratum*, it will offer an attractive and quick alternative for knocking down the expression of genes in *S*. *rostratum* without complicated genetic transformation methods or the requirement of a sequenced genome of *S*. *rostratum*.

During VIGS, an endogenous gene is degraded through a post-transcriptional gene silencing (PTGS) mechanism as the virus carrying a homologous gene in a viral vector spreads through the host plant [7]. At present, many VIGS vectors have been used to study gene function in plants, such as the potato virus X (PVX) [8], tobacco rattle virus (TRV) [6], and bean pod mottle virus (BPMV) [9]. The VIGS system has been established in many types of plants, including *N*. *benthamiana* [6], tomato [4], soybean [9], and potato [5]. Among all of the modified virus vector options, TRV is the most popular to facilitate the silencing of endogenous target genes in host plants because of the mild symptoms associated with its infection, and its wide range of potential plant hosts [10]. There are many well-defined methods of introducing TRV vectors into plants using *Agrobacteria*, such as leaf infiltration with a needleless syringe [6], using an artist's airbrush to spray plant leaves [4], vacuum infiltration of sprouts [11], and the use of agrodrench on the roots of young plants [12]. In order to obtain the best silencing efficacy, these various methods were used to infect different plants and tissues. For example, spraying (90% efficiency) *Agrobacterium* is more effective than infiltration (50% efficiency) in the induction of gene silencing in tomato leaves [4]. Agrodrench can be used for VIGS in very young seedlings where the leaf infiltration method is not possible [12]. Additionally, vacuum infiltration of *Agrobacterium* is far more effective than other strategies tested in *P*. *somniferum* [13].

In the present study, the genes for phytoene desaturase (*PDS*) and Chlorophyll H subunit (*ChlH*) of magnesium protoporphyrin chelatase were cloned from *S*. *rostratum* plants and used as VIGS reporter genes. Here, they were also used to establish a TRV-mediated VIGS system and optimize *Agrobacterium*-infiltration methods in *S*. *rostratum*. This study shows that TRV-mediated VIGS technology and tomato genomic sequences can be used in future studies that focus on gene function in *S*. *rostratum*.

## Material and Methods

### Plant material and growth conditions

*S*. *rostratum* seeds were obtained from Dr. Shouhui Wei, at the Institute of Plant Protection, Chinese Academy of Agricultural Sciences. *S*. *rostratum* seeds were germinated in flasks containing sterile water. The flasks were placed in a 25°C shaker for 24 h at 100 rpm. The seeds were transferred to a flat containing filter paper wetted with sterile water. After approximately 24 h, when the germinating seeds reached a length of 0.5–1 cm, they were subjected to sprout vacuum-infiltration. Treated sprouts were sown in pots and maintained in a growth chamber (23 ± 2°C, 20–30% RH) under a 16 h light/8 h dark cycle (600 lE/m2/s) and watered twice a week.

### Cloning of the *SrPDS* and *SrChlH* genes

The coding sequences (CDS) of *PDS* and *ChlH* genes of tomato and potato were obtained from GenBank and aligned to perform a homology analysis. Primers were designed based on these sequences. Total RNA was isolated from *S*. *rostratum* leaves using TRIzol Reagent (TIANGEN, Beijing, China) following the manufacturer’s instructions. Gene specific primer pairs were used to amplify *PDS* and *ChlH* from *S*. *rostratum* and the resulting PCR products were purified and sequenced.

### Plasmid construction

pTRV1 and pTRV2 VIGS vectors described in Liu *et al*. (2002) [4] were obtained from Dr. Savithramma Dinesh-Kumar at Department of Plant Biology University of California, Davis, Davis, California USA.

### pTRV2-*SrPDS* construction

To generate TRV2-*SrPDS*, a 479-bp *SrPDS* gene fragment was amplified from *S*. *rostratum* cDNA by RT-PCR using gene-specific primers (Forward: 5'-CGGGATCCCGGATAGGGTGACAGATGA-3' including a *Bam*HI restriction site. Reverse: 5'-CGGAATTCACACACTGAGCAGCGAACT-3' including an *Eco*RI restriction site). The RT-PCR product was digested with *Eco*RI and *Bam*HI and inserted into the *Eco*RI- and *Bam*HI-cleaved pTRV2 template plasmid.

### pTRV2-*SrChlH* construction

To generate TRV2-*SrChlH*, a 346-bp *SrChlH* gene fragment was amplified from *S*. *rostratum* cDNA by RT-PCR using gene-specific primers (Forward: 5'-CGGG ATCCGGGCAAGATGAGATGAAGTT-3' containing a *Bam*HI restriction site. Reverse: 5'-CGGAATTCTTTAGCAAGAAGACCAGGCT-3' containing an *Eco*RI restriction site). The PCR product is digested with *EcoR*I and *Bam*HI and inserted into the *Eco*RI and *Bam*HI-cleaved pTRV2 template plasmid.

### Sprout vacuum-infiltration and leaf infiltration methods

TRV1, TRV2-empty vector, and the *SrPDS* and *SrChlH* recombinant plasmids were introduced into *A*. *tumefaciens* strain GV3101 following the freeze–thaw method as previously reported [11]. Bacterial cells were grown at 28°C on Luria–Bertani (LB) medium with the appropriate selective antibiotics. For TRV infiltration solution preparation, a single colony was selected and used to inoculate 2 mL of LB liquid culture with the appropriate selective antibiotics. Bacterial cells were incubated overnight in 14-mL Falcon round bottom, polyethylene tubes with shaking at 200 rpm at 28°C. Then, 500 μl of the culture was used to inoculate a 20 mL of LB medium containing selective antibiotics, 10 mM M2-(N-Morpholino) ethanesulfonic acid (MES), and 20μM acetosyringone. The culture was incubated with shaking at 200 rpm at 28°C for 10 h. The bacteria cells were harvested by centrifugation and resuspended in infiltration buffer (10 mM MgCl_2_, 10 mM MES, 200 μM acetosyringone, pH 5.6) to an adjusted OD_600_ of 1.0 and left at room temperature for 3–4 h before infiltration. Each *Agrobacterium* culture containing TRV1 and TRV2-empty vector, TRV-*SrPDS*, or TRV-*SrCHLH* were mixed in a 1:1 ratio for the infiltration protocol. For sprout vacuum-infiltration, Silwet L-77 was added to the bacterial culture at a concentration of 0.05% (v/v) and immediately mixed well. The infiltration mixture and germinating seeds were placed in centrifuge tubes and then into a vacuum dryer. *Agrobacterium* was infiltrated into sprouts using a sprout vacuum-infiltration system [a vacuum dryer connected to a portable air compressor (GAST. INC)] set at a relative vacuum degree of -25 kPa. Vacuum pressure was maintained for approximately 30 s and then released rapidly to atmospheric pressure. This process was repeated once or twice for each infiltration event. Treated sprouts were then sown in pots of nutritional soil. For leaf infiltration, each *Agrobacterium* strain containing TRV1 and TRV2 vectors were mixed in a 1:1 ratio and infiltrated into the first true leaves of two-week-old plants or one week cotyledons with a 1 ml needleless syringe. Accumulation of virus in the freshly grown portions of the plants was detected by RT-PCR using the expression of the TRV coat protein (*CP*) as a marker (Forward primer: 5'-CCTTTATCCCTCTCCCTGACG-3'. Reverse primer: 5'-CCATCAAGTCAGCAGGACCG-3') two weeks after infiltration. Six to ten replicates were performed in each experiment and the experiment was repeated at least two times.

### RNA Extraction and Real-Time PCR

Total RNA was extracted from leaves of untransformed, silenced, and non-silenced (infiltrated with empty vector pTRV1 and pTRV2) plants using TRIzol reagent. cDNA was then synthesized from 0.5 μg of tRNA in 20 μL using the RNA PCR kit (Applied Biosystems) according to the manufacturer’s instructions with either oligo(dT) or TRV-RNA2 specific primers. Quantitative RT-PCR was performed using an ABI Prism 7900HT instrument and SYBR Green Master Mix (Applied Biosystems) with the following sequential protocol: 95°C for 5 min, followed by 40 cycles of 95°C for 15 s, 60°C for 30 s, 72°C for 60 s. Primers for PCR experiments were designed using the Primer 5.0 software and validated with Amplify v3.1 software. Primers were also designed to anneal outside the region targeted for silencing to ensure that only the endogenous gene would be amplified. Amplification using gene-specific primers for *Actin* (*ACT1*) served as an internal control. Standard dilution curves were performed for each gene fragment and all data were normalized to the *ACT1* transcript. The sequences of the primers are listed in S1 Table.

## Results and Discussion

### TRV can efficiently infect *S*. *rostratum*

The tobacco rattle virus (TRV) vector has been successfully used for gene silencing in a variety of plants, especially in the *Solanaceae* family members such as tomato [4], *Nicotiana benthamiana* [6], pepper [14], and eggplant [15]. Previous studies have shown that TRV can be transformed into the different tissues or organs of plants including the leaf [4], flowers [16], fruit [17], roots [18], and sprouts [11] of *Solanaceous* species. We hypothesized that as a member of the *Solanaceae* family, *S*. *rostratum* might also be susceptible to TRV-induced gene silencing.

**Fig 1:**
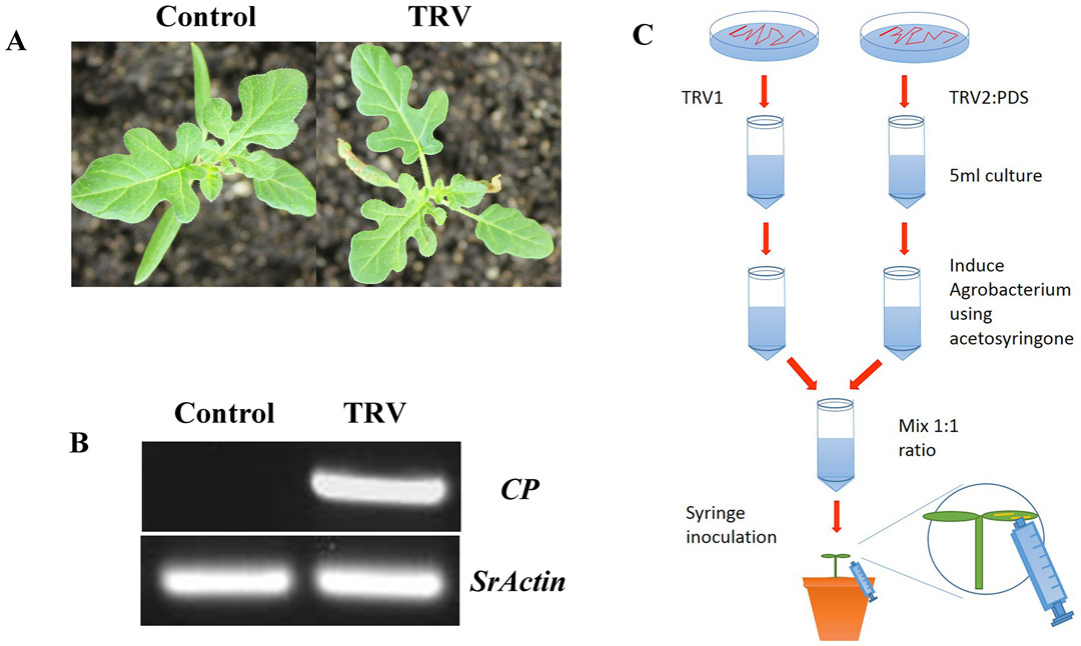
TRV can infect *S*. *rostratum*. (A) *S*. *rostratum* plants infiltrated with *Agrobacteria* carrying the empty TRV vector were imaged two weeks after inoculation. (B) RT-PCR using TRV *CP*-specific primers and *S*. *rostratum*; *ACT1* expression from cDNA samples of TRV-infiltrated and control *S*. *rostratum* plants. (C) A schematic depiction of VIGS treatment in *S*. *rostratum* plants using a needleless syringe for infiltration method.

Taking into account that effective viral infection is a prerequisite for VIGS in plants, we first tested whether the TRV virus can efficiently infect *S*. *rostratum*. Plants were inoculated using a mixture of *Agrobacterium tumefaciens* (GV3101) cultures containing pTRV1 and pTRV2 constructs in a 1:1 ratio that was syringe-infiltrated into the cotyledon of the plants one week after germination. Plants infiltrated with *Agrobacterium* cultures were used as a control (Fig 1A). Total RNA was extracted from an upper, un-infiltrated true leaf of inoculated plants after two weeks for cDNA preparation to assess the spread of virus. Random primers were used to generate the cDNA template for the amplification of the coat protein (*CP*) gene of the TRV virus. The *CP* gene was detected in TRV-infected plants but not in control plants (Fig 1B). The *ACT1* gene was used as an internal control in all RT-PCR assays. The sequencing of the PCR product of the expected *CP* gene was consistent with the fragments included in the TRV2 plasmid (data not shown). However, we observed no visible virus symptoms in TRV-infected plants when compared to the wild type, suggesting that the TRV symptoms did not interfere with the gene silencing phenotype in *S*. *rostratum* plants. These results show that recombinant TRV can efficiently infect *S*. *rostratum* plants. We next tested whether TRV can silence the endogenous genes in *S*. *rostratum* plants.

### Cloning of the reporter genes *PDS* and *ChlH* from *S*. *rostratum* for VIGS

Before we tested the VIGS effects in *S*. *rostratum*, reporter genes needed to be selected. The *PDS* and *ChlH* genes have previously been shown to be good candidates for reporter genes in VIGS experiments [4, 6, 15]. *PDS* silencing in *N*. *benthamiana* inhibits carotenoid biosynthesis, causing the plants to exhibit a photo-bleaching phenotype [19]. The *ChlH* gene encodes the H subunit of magnesium protoporphyrin chelatase [20], an enzyme involved in chlorophyll biosynthesis. Reduction or absence of magnesium protoporphyrin chelatase in plants results in yellow-colored leaves because of this reduction in chlorophyll synthesis [15, 21].

**Fig 2:**
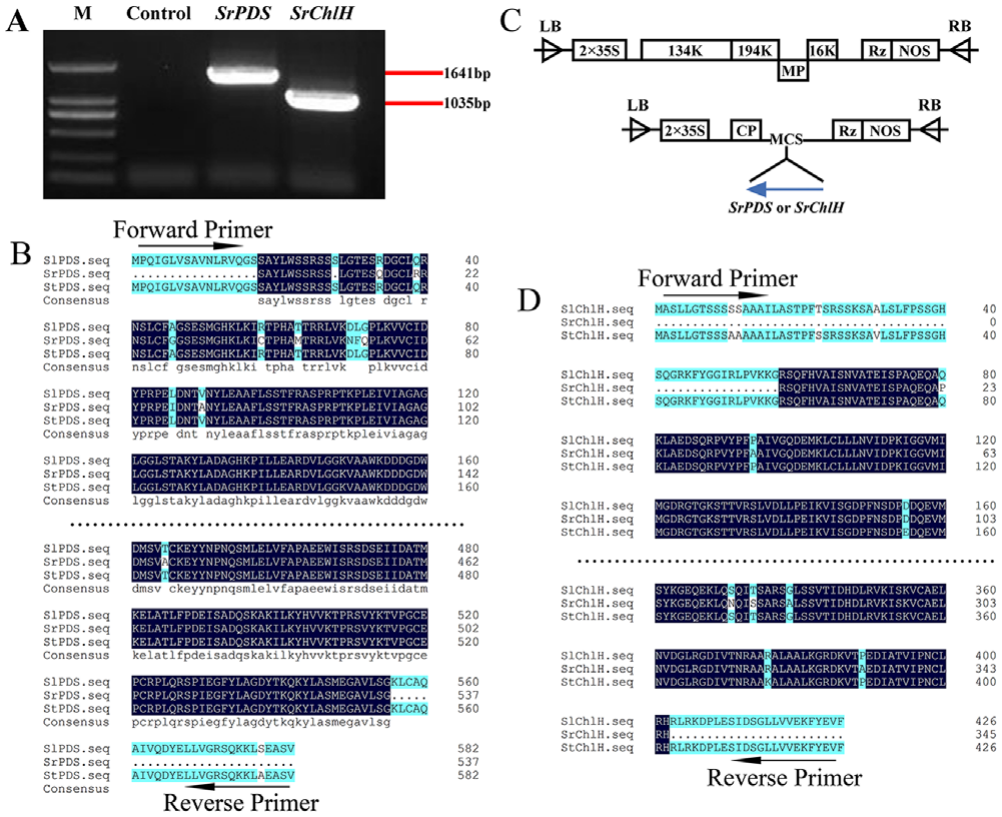
Cloning of *SrPDS* and *SrChlH* genes from *S*. *rostratum* and the construction of TRV-*SrPDS* and TRV-*SrChlH*. (A) Amplification of *SrPDS* and *SrChlH* from *S*. *rostratum* plants. Primers for *PDS* and *ChlH* were designed from conserved regions of *PDS* or *ChlH* based on the alignment of tomato and potato CDS sequences (red arrows in panel). *S*. *rostratum* cDNA was used as a template to amplify *SrPDS* (479-bp) and *SrChlH* (346-bp) PCR products. An RT reaction without reverse transcriptase served as a control. (B, D) Amino acid sequences of PDS and ChlH from tomato and potato were used in an alignment analysis of *S*. *rostratum*. (C) Diagrams of TRV-*SrPDS* and TRV-*SrChlH*. The amplified *S*. *rostratum* gene fragments were inserted into pTRV2 to obtain the pTRV-*SrPDS* and pTRV-*SrChlH* silencing vectors.

It was difficult for us to directly clone *PDS* and *ChlH* genes from *S*. *rostratum* owing to the lack of effective gene information on this species in GenBank. However, taking into account that both tomato and potato share very high sequence similarity and belong to the same family as *S*. *rostratum*, we hypothesized that the three plants would have similar genomic sequences. We first downloaded the sequences of *ChlH* and *PDS* genes of tomato and potato from GenBank and performed a homology analysis. We found that there is more than 95% similarity at the gene level between the tomato and potato for both *PDS* and *ChlH*. Using the conserved homologous regions of these genes, two pairs of primers were designed to clone *ChlH* and *PDS* from *S*. *rostratum*, respectively. Clear bands were obtained by PCR for each gene(Fig 2A), and they were excised for further analysis. Sequencing results indicated that the cloned CDS sequences of *SrChlH* and *SrPDS* shared 94.58% and 97% identity with the tomato *ChlH* and *PDS* genes, respectively (Fig 2B and 2D). The cloned fragments of these genes were sufficient to be used as silencing reporters of VIGS in *S*. *rostratum*.

### Silencing of *SrChlH* and *SrPDS* genes in *S*. *rostratum*

**Fig 3:**
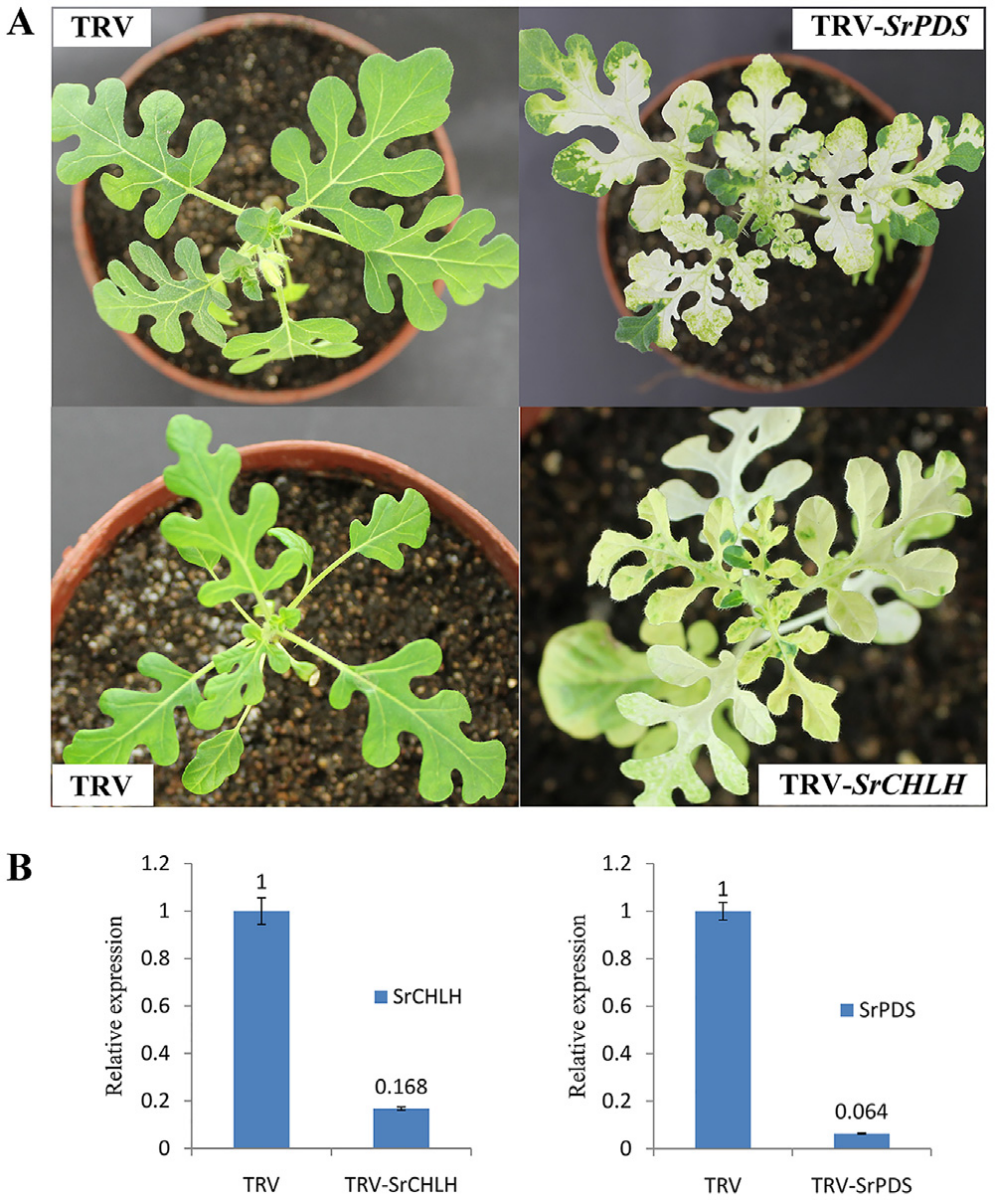
Silencing of *PDS* and *ChlH* genes in *S*. *rostratum* plant seedlings. (A) The *SrPDS* and *SrChlH* silencing phenotypes in *S*. *rostratum* plants. *Agrobacterium* containing TRV alone, pTRV2-*SrPDS* or pTRV2-*SrChlH* was infiltrated into the first true leaves of *S*. *rostratum* seedlings together with pTRV1. Two weeks after inoculation, photo-bleaching was observed in the *PDS*-silenced plants and yellowing was observed in the *ChlH*-silenced plants. Control plants inoculated with TRV alone were green in color. (B) RT-PCR analysis of mRNA levels of *SrPDS* and *SrChlH* in TRV control and *PDS* or *ChlH* gene-silencing plant. Error bars show +/-SD.

We tested whether the TRV clones could induce endogenous gene silencing in *S*. *rostratum* plants. The fragments of the 346-bp *SrChlH* and 479-bp *SrPDS* genes were cloned from *S*. *rostratum* and inserted into the pTRV2 vector to produce the pTRV-*SrChlH* and pTRV-*SrPDS* vectors(Fig 2C). A mixture of *Agrobacterium* cultures containing pTRV2-*SrPDS* or pTRV2-*SrChlH* and pTRV1 were infiltrated onto the first true leaves of two-week-old *S*. *rostratum* plants. Two weeks after *Agrobacterium* infiltration, *S*. *rostratum* plants infected with pTRV-*SrPDS* or pTRV-*SrChlH* exhibited a photo-bleached or yellow leaves phenotype in the upper leaves of plants, respectively (Fig 3A). Although most of the *S*. *rostratum PDS*-silenced plants showed a severe phenotype with complete photo bleaching in all newly emerging leaves, some *S*. *rostratum PDS*-silenced plants exhibited a less severe phenotype of a patchy pattern of photo-bleaching (data not shown). This suggests there is some variation in the extent of gene silencing, which may be due to subtle differences between individual plants or the uptake of the TRV constructs after infiltration.

To confirm the *SrPDS or SrChlH* suppression at the molecular level, primers specific to the *SrChlH* or *SrPDS* genes outside the region targeted for silencing were designed for real-time PCR. The reduction in *SrChlH* and *SrPDS* transcripts in infected plants was 84% and 94%, respectively, of that in the control plants (Fig 3B). The level of *ACT1* transcript was similar in tissues infected with TRV-*SrPDS*, TRV-*SrCHLH*, and TRV-empty vector constructs. The effective VIGS of the reporter genes in *S*. *rostratum* seedlings using TRV constructs suggests that other genes could also be targeted for silencing in a similar manner.

### The photo-bleached phenotype of *SrPDS-*silencing can persist in flowers and fruit in the infected *S*. *rostratum* plants

**Fig 4:**
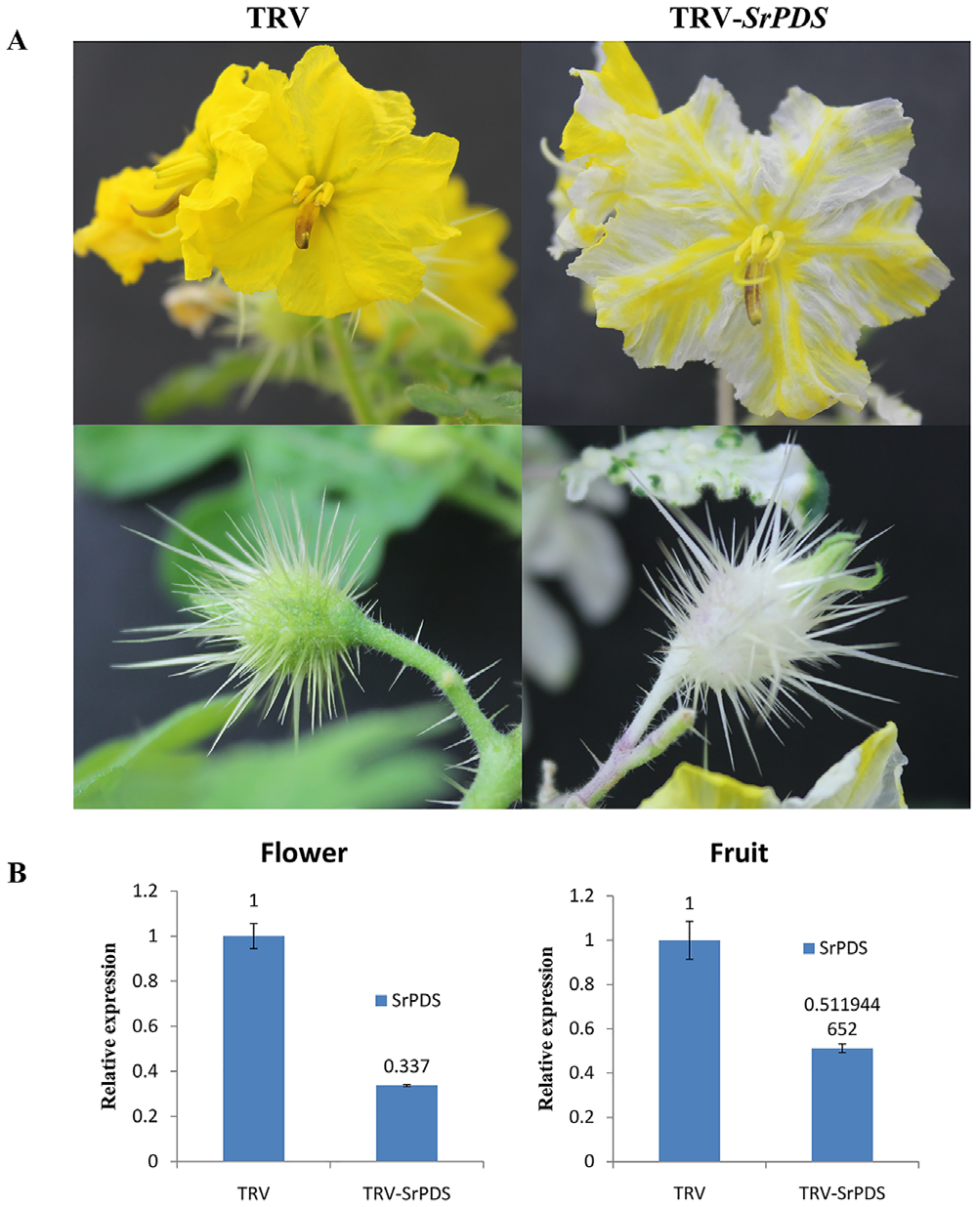
Silencing of *PDS* genes in the flowers and fruit of *S*. *rostratum*. (A) The phenotype of *SrPDS* silencing in *S*. *rostratum* flowers and fruit. *Agrobacterium* containing TRV alone or TRV-*SrPDS* was co-infiltrated with pTRV1 into the first true leaves of *S*. *rostratum* seedlings together. Photo-bleaching was observed in the flowers and fruit that developed 44–45 days after infection on the *PDS*-silenced *S*. *rostratum* plants. Control plants inoculated with TRV alone produced yellow flowers and green fruit. (B) Real-time RT-PCR analysis of *SrPDS* transcript levels in the flowers and fruit of the TRV control and TRV-*SrPDS* plants. Error bars show +/- SD.

In order to test whether gene silencing can be transported to the reproductive organs of *S*. *rostratum* plants, *SrPDS*-silenced *S*. *rostratum* plants were kept in suitable growth conditions for 45–55 days after infiltration until flowers and fruit developed. PDS-silenced plants developed some yellow flowers with white spots and white fruit compared to the yellow flowers and green fruit of control plants (Fig 4A). RT-PCR showed that *SrPDS* transcript levels in the white flower of SrPDS-silenced plants were dramatically reduced by 73%, as compared to that of yellow flowers of control plants. Likewise, *PDS* transcript levels in the white fruit dropped by 84% (Fig 4B). Taken together, these data show TRV can induce gene silencing in the reproductive organs of *S*. *rostratum* plants. This method provides a long-term, systematic VIGS effect in *S*. *rostratum*. I think it may also be important to point out that these plants were infiltrated in early leaves, showing that not only is the transfection long lasting, but it also can affect multiple tissue/organs. This is impactful because the leaf infiltration is quite easy to do, and can provide many different avenues of investigation in plant biology through its effect on the whole plant.

### Optimization of the TRV infection method in *S*. *rostratum*

**Fig 5:**
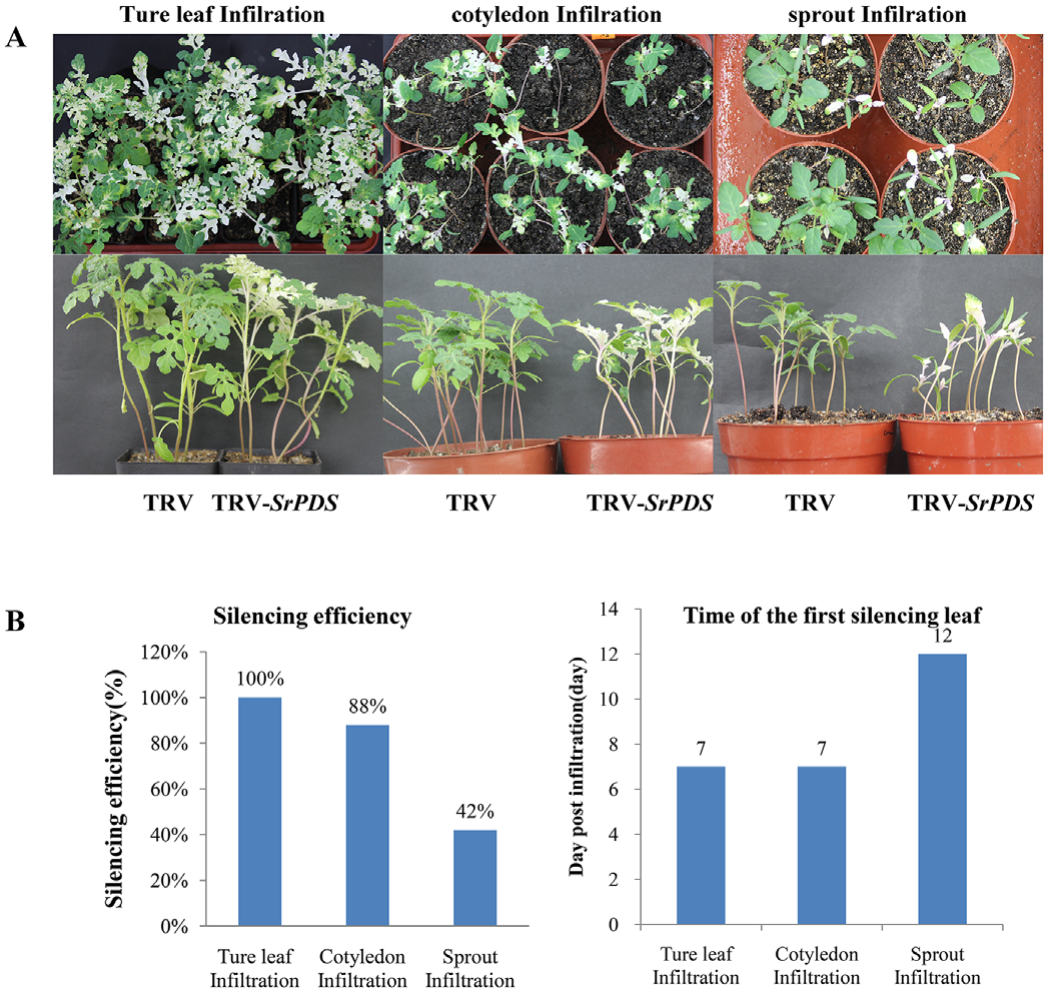
Comparison of agroinoculation methods in *S*. *rostratum*. (A) Effects of three agroinoculation methods. *Agrobacterium* cultures containing pTRV1 and pTRV2 alone (TRV) or TRV2-*SrPDS* were mixed in a 1:1 ratio and infiltrated into the true leaves and cotyledons of *S*. *rostratum* with a needleless syringe, or into sprouts using a vacuum. (B) Efficiency of *SrPDS* gene silencing of *S*. *rostratum* plants and the first appearance of *SrPDS* silencing in leaves for each treatment. Silencing efficiency was assessed 14 days after TRV inoculation. The data are representative of three independent experiments, with N = 100 for each experiment.

In order to determine the best method of TRV infiltration of *S*. *rostratum* plants, true leaf infiltration, cotyledon infiltration, and sprout vacuum infiltration were selected for comparison. Each method was performed in three repeated experiments, each composed of 100 plants. *Agrobacterium* strains containing TRV1 and TRV2-*SrPDS* were mixed in a 1:1 ratio and applied directly onto the first true leaves or cotyledons of young plants with a 1 ml needleless syringe. In addition, sprouts were inoculated using vacuum infiltration (Fig 5A). The frequency of VIGS was determined by the number of plants that showed the silencing phenotype (photo-bleaching) two weeks after inoculation with TRV2-*SrPDS*. We observed silencing efficiencies of 100%, 88%, and 42% in the three infiltration methods (true leaf infiltration, cotyledon infiltration, and sprout vacuum infiltration), respectively. We also evaluated the time it took for the first instance of photo-bleaching to appear after inoculation with TRV2-*SrPDS* (Fig 5B). Results of leaf and cotyledon infiltration showed that photo-bleaching was first observed on the newly developed leaves of *S*. *rostratum* plants 7 days after inoculation. Sprout infiltration VIGS effects were delayed by 5 days compared to the other methods, and showed a 46–58% reduction in the silencing frequency in *S*. *rostratum* plants (Fig 5B). The delay in silencing may be due to the length of time required for the virus infection to develop, or for the silencing signal to move from the site of infection on the sprout to the leaves of the plant. Additionally, sprouts inoculated with *Agrobacteria* generally grow slowly, which could also account for the delay in the presentation of silencing phenotypes. Our results are evidence that of the three methods tested, first true leaf infiltration is the best method for gene silencing in *S*. *rostratum* plants.

## Conclusion

Virus-induced gene silencing methods that do not rely on stable plant transformation offers a tremendous advantage for gene function analysis [7, 10, 22]. In the present study, we demonstrated that recombinant TRV can infect *S*. *rostratum* plants and can be used to efficiently silence genes, as we showed using the *SrPDS* and *SrChlH* genes as silencing reporters. In order to clone *SrPDS* and *SrCHLH* for their use as reporter genes, we used tomato gene sequences to design primers and obtain *SrPDS* and *SrCHLH* fragments from *S*. *rostratum* cDNA amplification. This confirms that the tomato genome can be used to identify and silence homologous genes in *S*. *rostratum* by VIGS. Using the TRV-based VIGS system, we silenced *SrPDS* in the leaves, flowers, and fruit of *S*. *rostratum* plants, further demonstrating that TRV can effectively move to other tissues and organs from infiltrated leaves. In order to determine the best agroinoculation method for *S*. *rostratum* plants, we assessed three methods of inoculation based on silencing efficiency and the time when the first *SrPDS* silencing appeared in leaves. We tested the infection efficiency using leaf, cotyledon, and sprout infiltration methods and found that leaf infiltration had the highest level of gene silencing in *S*. *rostratum*. These results provide evidence that the first true leaf-infiltration method is the best for obtaining high infection efficiency and faster silencing as compared to cotyledon and sprout infiltration. Therefore, TRV-mediated VIGS can be used for future large-scale, functional genomics in *S*. *rostratum* plants.

## Supporting Information

S1 Table Primers list of Real-time PCR for *SrdPDS* and *SrdCHL* gene. (DOCX)
