# Supplementary material for: Efficient Virus-Induced Gene Silencing in Solanum rostratum
Source: PLoS One. 2016 Jun 3;11(6):e0156228. doi: 10.1371/journal.pone.0156228 (PMC4892644; doi:10.1371/journal.pone.0156228)
Supplement: S1 Table — (DOCX) [file pone.0156228.s001.docx]

**Sl Table1: Primers list of Real-time PCR for *SrdPDS* and *SrdCHL* gene**

| Primer name | Primers |
| --- | --- |
| SrdPDSForward | 5'-GTGGAAAGGTAGCTGCATGG-3' |
| SrdPDSReverse | 5'-CACTGCAATCGATCATTAATCCC-3' |
| SrdCHLForward | 5'-AAGAAGGAGAACTTAGGCCAC-3' |
| SrdCHLReverse | 5'-CTCTTTCCTCGACGATCTTCAC-3' |
| ActinForward | 5'-CAGCAGATGTGGATCTCAAA-3' |
| ActinReverse | 5'-CTGTGGACAATGGAAGGAC-3' |
